# Medical Practice by Commercial Companies

**Published:** 1900-03-31

**Authors:** 


### MEDICAL PRACTICE BY COMMERCIAL COMPANIES.

The Companies' Act Amendment Bill which has been introduced into the House of Commons by Mr. Ritchie, contains a clause the intention of which is to prevent companies from conducting medical, dental, or midwifery practice. It runs as follows: "3. It shall be unlawful for a company to carry on the profession or business of a physician, surgeon, dentist, or midwife; and if any company contravene this enactment, it shall be liable, on summary conviction, to a fine not exceeding five pounds for every day during which the contravention happens." Lest the Government should feel tempted to drop this clause in view of the opposition which it may arouse in interested quarters, a memorial to the President of the Board of Trade has been drawn up by the members of the Council of the British Medical Association, urging the necessity of some such restriction being placed upon the doings of commercial companies.

